# Whole-body computed tomography versus conventional skeletal survey in patients with multiple myeloma: a study of the International Myeloma Working Group

**DOI:** 10.1038/bcj.2017.78

**Published:** 2017-08-25

**Authors:** J Hillengass, L A Moulopoulos, S Delorme, V Koutoulidis, J Mosebach, T Hielscher, M Drake, S V Rajkumar, B Oestergaard, N Abildgaard, M Hinge, T Plesner, Y Suehara, K Matsue, N Withofs, J Caers, A Waage, H Goldschmidt, M A Dimopoulos, S Lentzsch, B Durie, E Terpos

**Affiliations:** 1 Department of Hematology and Oncology, University Hospital of Heidelberg, Heidelberg, Germany; 2 Department of Radiology, German Cancer Research Center, Heidelberg, Germany; 3 First Department of Radiology, National and Kapodistrian University of Athens, School of Medicine, Areteion Hospital, Athens, Greece; 4 Department for Biostatistics, German Cancer Research Center, Heidelberg, Germany; 5 Division of Endocrinology, Mayo Clinic, Rochester, MN, USA; 6 Division of Hematology, Mayo Clinic, Rochester, MN, USA; 7 Department of Hematology, Odense University Hospital, University of Southern Denmark, Odense, Denmark; 8 Department of Hematology, Vejle Hospital and University of Southern Denmark, Vejle, Denmark; 9 Division of Hematology and Oncology, Department of Medicine Kameda Medical Center, Kamogawa-shi, Japan; 10 Department of Nuclear Medicine, CHU de Liège, Belgium; 11 Department of Hematology, CHU de Liège, Belgium; 12 Department of Hematology, St Olavs Hospital, Norwegian University of Science and Technology, Trondheim, Norway; 13 Department of Clinical Therapeutics, National and Kapodistrian University of Athens, School of Medicine, Alexandra General Hospital, Athens, Greece; 14 Department of Medicine, Division of Hematology/Oncology, Columbia University Medical Center, New York City, NY, USA; 15 Cedars-Sinai Samuel Oschin Cancer Center, Los Angeles, CA, USA

## Abstract

For decades, conventional skeletal survey (CSS) has been the standard imaging technique for multiple myeloma (MM). However, recently whole-body computed tomography (WBCT) has been implemented into the diagnostic criteria of MM. This analysis compares sensitivity and prognostic significance of WBCT and CSS in patients with smoldering MM (SMM) and MM. Fifty-four of 212 patients (25.5%) had a negative CSS and a positive WBCT for osteolytic lesions (*P*<0.0001). Of 66 patients with SMM based on CSS, 12 (22.2%) had osteolytic lesions on WBCT. In comparison, WBCT failed to detect some bone destructions in the appendicular skeleton possibly due to limitations of the field of view. Presence of lytic bone lesions in WBCT was of borderline prognostic significance (*P*=0.051) for SMM patients, with a median time to progression of 38 versus 82 months for those without bone destructions. In conclusion, WBCT identifies significantly more sites of bone destruction than CSS. More than 20% of patients with SMM according to CSS have in fact active MM detectable with WBCT. On the basis of this and other studies, WBCT (either computed tomography (CT) alone or as part of a positron emission tomography-CT protocol) should be considered the current standard for the detection of osteolytic lesions in MM.

## Introduction

For decades, conventional skeletal survey (CSS) has been the standard imaging technique for the detection of myeloma bone disease.^1, 2^ It consists of conventional X-rays of multiple skeletal sites (skull, spine, pelvis, chest, femora and humeri). CSS was the basis for the Durie/Salmon staging system and for the guidelines of the International Myeloma Working Group (IMWG) as of 2003.^3, 4^ Earlier publications had shown, however, that the sensitivity of conventional X-rays for bone damage assessment was limited, as changes were not detected until at least 30–50% of bone mass was destroyed.^5^ Computed tomography (CT), with its ability to provide three-dimensional information of the examined region is as expected more sensitive than projection X-ray studies. Since bone is a high-contrast organ (mineralized bone versus fat-containing bone marrow), CT radiation doses needed for optimal skeletal imaging are lower than those required for differentiating soft tissue pathologies. Therefore, dedicated low-dose, whole-body CT protocols have been developed for skeletal imaging. Several studies have already investigated the use of whole-body CT (WBCT) in patients with monoclonal plasma cell disorders, showing that WBCT is more sensitive for detecting skeletal lesions than plain X-ray films.^6, 7, 8, 9^ Therefore, more modern cross-sectional imaging techniques have been implemented into the updated guidelines of the IMWG for the diagnosis of multiple myeloma (MM).^10^ However, concerns have been raised that due to the higher sensitivity of WBCT, bone changes might be detected earlier but might not yet be clinically relevant. In this setting, patients would be classified as having symptomatic myeloma but would not benefit from earlier initiation of treatment.

This study aimed to both, compare the sensitivities of WBCT and CSS for the detection of skeletal lesions, and to determine whether additional lesions detected by WBCT are of prognostic relevance. Given logistical challenges in identifying patients who have been examined with both CSS and WBCT almost simultaneously, previous studies comparing both techniques have mostly included comparatively few patients. Further, studies investigating the prognostic significance of earlier detection of myeloma bone disease is lacking. To address these deficiencies, the IMWG decided to perform a retrospective, international multicenter analysis of patients with smoldering multiple myeloma (SMM) who had, for any reason, been examined with both conventional X-ray and WBCT within 30 days.

## Patients and methods

Inclusion criteria for patients in the current analysis were histologically proven MM with WBCT and CSS obtained within 30 days. Furthermore, the mentioned questionnaire had to contain all of the required clinical information to allow for disease staging. For the comparison of CSS and WBCT pretreated patients were also included; for the analysis of the prognostic significance of imaging findings only untreated patients.

### Data acquisition

For the current retrospective analysis, we collected data, CSS and WBCT studies from a total of 283 patients from eight different centers worldwide. From this cohort, 212 patients with myeloma (66 with smoldering myeloma) fulfilled the inclusion criteria. Of this 212 patients, 159 were untreated at time point of imaging. A list of contributing centers can be found in the Supplementary Data (Supplementary Table 1). Compact discs with pseudonymized imaging data of WBCT and CSS were sent to the organizing center and uploaded to a dedicated workstation running a local installation of Osirix MD (Pixmeo SARL, Bernex, Switzerland), a commercially, FDA-approved picture archiving and communication system (PACS) that provides all tools for viewing and analyzing radiological images, equipped with a monitor certified for medical use. Furthermore, questionnaires were completed by each of the centers collecting clinical data of the patients.

Image analysis was performed in consensus reading by three experienced radiologists (LAM, SD, VK) blinded to the clinical data of the patients. WBCT and CSS studies were read several months apart, so that the readers were unable to attribute WBCT and CSS images of one patient to each other. Lesions were classified as ‘definitely present’, ‘probably present’, ‘probably absent’ and ‘definitely absent’ for each lesion and in the following locations: Skull, cervical spine, thoracic spine, lumbar spine, sacral bone, iliac bones, sciatic bones, pubic bone, sternum, left rib cage (Ribs L), right rib cage (Ribs R), left scapula, right scapula, left clavicle, right clavicle, left humerus, right humerus, left femur and right femur, respectively. Furthermore, the presence of hyperdensities, either focal or diffuse, in the medullary cavity of the femora or humeri was recorded but was not analyzed for the present comparative study.

### Patient characteristics available data

Median age of the patient group was 66.1 years (age range 40.2–91.9 years). Median time interval between WBCT and CSS was 1 day −29 to 30 days. Patient characteristics are shown in Table 1.

### Definitions and limitations of collected data

Some osseous locations were not included in CSS in some centers. In such cases, only those osseous locations having valid measurements for both WBCT and CSS methods were considered.

SMM and MM were defined according to the current guidelines of the IMWG.^10^ Bone disease-defining CRAB criteria were based on lytic lesions results from CSS.

### Statistical analysis

For statistical tests, ‘definitely present’ and ‘probably present’ entries were merged and counted as lytic lesions. WBCT and CSS were tested for difference in detection sensitivity with the exact McNemar test including odds ratio and 95% confidence interval. The 95% confidence interval for difference in paired proportion was calculated. Time to progression (TTP) of disease was calculated from time of CSS onwards. Log-rank test was used to compare TTP between groups. Aalen–Johansen estimator was used to estimate the cumulative incidence of progression accounting for death as competing event. Analysis was performed with statistical software R 3.3.0 (Vienna, Austria).

## Results

### Comparison of different techniques in the entire patient cohort

A total of 212 patients were included in the analysis for comparison of the WBCT and CSS imaging techniques. In 103 patients (48.6%), no lytic bone lesions were detected with either technique, and in 43 patients (20.3%) lesions were detected with both CSS and WBCT. In 12 patients (5.7%), lytic lesions were seen in CSS but not by WBCT. In 54 patients (25.5%), lytic bone lesions were seen by WBCT but not on CSS (odds ratio 4.50 (2.38–9.24); *P*<0.0001).

Table 2 shows the numbers of patients with definitely or probably positive, as well as probably or definitely negative results in CSS and WBCT, respectively.

### Detection differences of CSS and WBCT for lytic bone lesions based on anatomic location

**Figure 1:**
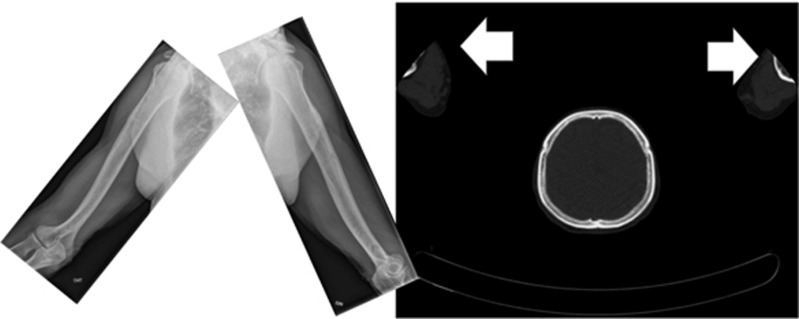
X-rays (left) and CT (right) showing the limited field of view for some CT protocols in which the arms had been placed above the head. In such cases, the elbows protruded outwards, and as a result, the distal humeri were either ‘cut off’ or lay in the field periphery where the image quality of many CT scanners is limited.

**Figure 2:**
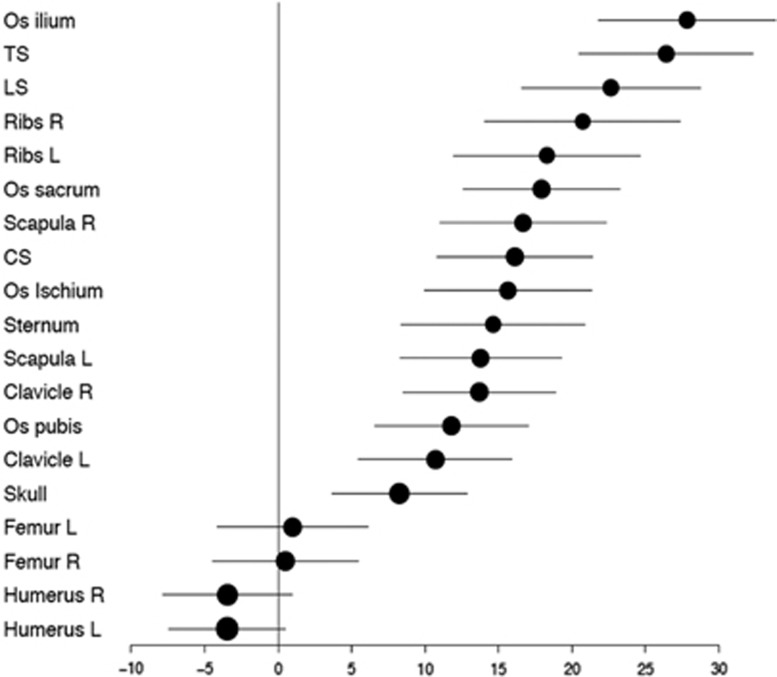
Detection differences (including 95% confidence intervals) between WBCT and X−ray for the whole patient group in %. CS, cervical spine; LS, lumbar spine; TS, thoracic spine.

The difference in detection sensitivity for WBCT and CSS was dependent on the location of the lesions in the skeleton. WBCT was overall superior, particularly in the axial skeleton. CSS detected slightly more bone destruction only in the humeri, a fact which in some cases may be attributable to limitations in the field of view with WBCT (Figure 1). Of the 12 patients in whom WBCT was negative but there was suspicion of lytic lesions in CSS, in only 5 patients were lesions defined as ‘definitely present’. In six remaining patients, osteolytic lesions were determined to be ‘probably present’, while in one patient, lesions in CT were counted as ‘probably absent’ and as ‘probably present’ in CSS. In total, these were 21 lytic lesions, of which 11 were localized to the long bones (clavicle, femur, humerus, ribs), and 10 in the axial skeleton (skull, spine, pelvis). Detection rates depending on location in the skeleton are shown in Figure 2 and Supplementary Table 2.

### SMM patients

After review of the clinical data provided by the contributing centers and the results of CSS, 66 patients were identified as having SMM according to the 2003 IMWG guidelines, with 54 of the 66 being untreated. Those 54 patients were included in the current analysis.

In 42 of the untreated SMM patients (77.8%), both CSS and WBCT techniques did not show any lesions. In 12 patients, however, WBCT identified osteolytic lesions (22.2%), while CSS was by definition negative (*P*=0.0005) (Table 3).

**Figure 3:**
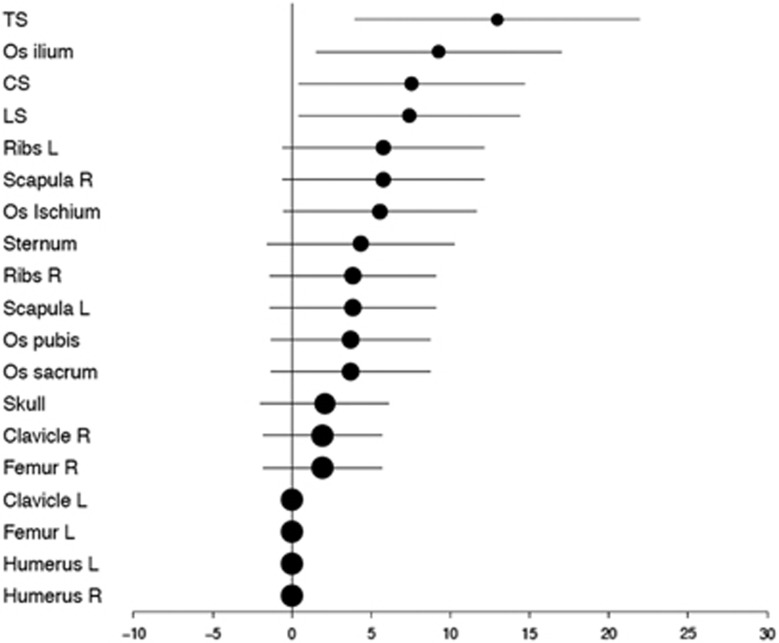
Detection differences (including 95% confidence intervals) between WBCT and CSS in patients with SMM in %.

Sensitivity of WBCT was most significantly superior to CSS in the spine and pelvis as shown in Figure 3. Those patients in whom CSS showed bone destructions in the appendicular skeleton (partly missed by WBCT) did, by definition, not fulfill the criteria of SMM and were not included in this analysis.

In comparing clinical parameters in patients with SMM, we found the patients who had bone destruction present when assessed by WBCT had significantly higher serum creatinine values (*P*=0.03), but were, by definition, not beyond the limit necessary for the initiation of anti-myeloma treatment. No other significant differences between the groups were identifiable (for clinical questionnaire see Supplementary Appendix).

Osteoporosis, as defined by standard radiological criteria, was detected by both techniques in 14 SMM patients. In 12 patients, osteoporosis was detected by WBCT only, and in 12 patients, osteoporosis was detected only on CSS (odds ratio 12.0; *P*=0.003). In four patients, WBCT showed osteoporosis with fracture. In these patients, CSS showed neither osteoporosis nor a fracture.

### Prognostic significance of WBCT findings in patients with SMM

**Figure 4:**
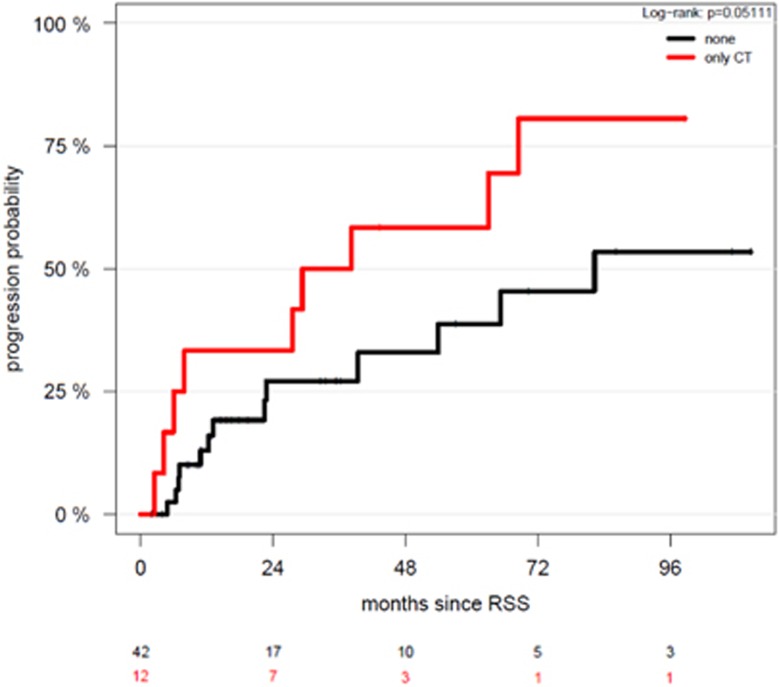
Cumulative incidence of progression to symptomatic MM in patients with or without osteolytic lesions by WBCT.

The 12 SMM patients who had lytic bone lesions on WBCT had a higher probability of progression to symptomatic myeloma than those with no lytic bone lesions in WBCT (log-rank *P*=0.05) (Figure 4). However, no prognostic significance for overall survival was identifiable. None of the SMM patients progressed within the first 2 months after imaging. SMM patients with lytic bone lesions identified by WBCT had a median TTP of 38 months compared to 82 months in patients without lytic bone lesions, and a 2-year progression-free survival of 58% versus 33%, respectively. Osteoporosis was not of prognostic significance. However, if a patient with SMM had an osteoporotic fracture, his/her risk for progression was be increased (hazard ratio (HR) 2.03; confidence interval 0.57–7.24), although not significantly, possibly due to the small number of patients.

### Prognostic significance of WBCT findings in patients with MM

In the 79 untreated MM patients, 48 (60.8%) showed osteolyses in CT while in 40 (50.6%) patients osteolyses were found in CSS. A difference between both imaging techniques was found with CT positivity and CSS negativity in 16 (20.3%) and CSS positivity with CT negativity in 8 (10.1%) of MM patients, respectively. No prognostic significance was found for the presence of lytic bone lesions identified by either technique (progression-free survival: *P*=0.3 and OS: *P*=0.4).

## Discussion

Due to the expected higher sensitivity of CT over CSS, several centers worldwide have already switched from CSS to WBCT for examining myeloma bone disease, using either CT alone or as part of a positron emission tomography (PET)-CT protocol. The latest guidelines of the IMWG also recommend initiating therapy if CT imaging shows one or more lytic bone lesions.^10^ However, concern has been raised that detection of bone lesions earlier might trigger systemic treatment at a time when it is not yet necessary, thus causing unnecessary potential treatment side effects or complications to patients.

Therefore, this study sought to determine how frequently bone lesions are detected by WBCT in patients who, per former criteria, would not have needed treatment, and to learn whether patients with positive WBCT but negative CSS are at increased risk for earlier MM progression. Since, owing to radiation dose concerns, WBCT and CSS are rarely performed at the same time, eight centers within the IMWG agreed to retrospectively collect imaging and clinical data from patients in whom WBCT and CSS had been performed for any reason (for example, CSS and PET-CT, CT as new standard and CSS as part of a clinical trial and so on) within 30 days. Another benefit of performing this analysis was that different imaging protocols would be available for comparison, which would aid in establishing minimal requirements for WBCT protocols. Guidelines on this topic will be published separately by the participating radiologists.

The current multicenter study investigating 212 evaluable patients confirms that in 20–25% of patients with negative CSS, WBCT will detect destructive bone lesions. This is compatible with earlier but smaller studies, which revealed that conventional CT imaging is superior to imaging by plain X-ray in 51, 32, 18 and 35 patients with MM, respectively.^7, 9, 11, 12^ Horger *et al.*^13^ were the first to perform a feasibility study comparing a WBCT protocol to CSS in 100 patients with Monoclonal Gammopathy of Undetermined Significance and partly pretreated patients with MM, which showed that CT provided an adequate detection rate of lytic bone lesions and was useful to determine fracture risk. In that study, however, no plain film X-rays were available for comparison. Kröpil *et al.* compared findings of WBCT and CSS in 29 partially pretreated patients with MM in Durie and Salmon stages I–III. In 18 patients, 97 lesions were detected exclusively by WBCT.^8^ Further, in a heterogeneous cohort of 52 patients with monoclonal plasma cell disorders, Wolf *et al.*^6^ found more lytic bone lesions by WBCT compared to CSS in 12 patients (23%), a similar rate to that determined in our study. Finally, a recent study from Mayo Clinic investigated 188 patients with SMM with PET-CT. In 122 evaluable patients, 16 (13%) showed PET positivity and lytic bone lesions in the CT portion of the PET-CT.^14^ False negative results of WBCT occurred particularly in the upper extremities, and scans that were performed for attenuation correction in PET-CT and/or with the arms were placed above the head. In the former case, the tube current is very low, that is, even lower than in a dedicated skeletal low-dose CT. The reason is that the diagnostician focusses mainly on reading the PET study and uses the CT scans solely for the attenuation correction of the PET raw data, and to attribute an Fluorodeoxyglucose-avid focus to an anatomical location. In the latter case, positioning the arms above the head, with the elbows pointing outside, the distal humeri, elbows and proximal forearms will typically lie outside the field for which the CT scan is reconstructed. The result will be that the X-rays are attenuated not only by the objects within the reconstructed field of view but also by structures outside of it. This causes malcalculations during the image reconstructions and to a significant degradation of the image quality particularly in the periphery of the field of view. Typically, the density values will be too high, and there will be additional streak artefacts and also increased image noise.

Since generalized osteoporosis is difficult to detect by standard plain films and is also subject to a variety of influences including aging, osteoporosis *per se* is no longer a criterion for myeloma bone disease in the current IMWG guidelines.^10^ In our cohort, WBCT was significantly superior to CSS for detecting osteoporosis, particularly for the identification of osteoporotic fractures, as in four patients, osteoporotic fractures were invisible by CSS. Thus although clinically relevant, a radiological diagnosis of osteoporosis in the absence of lytic bone lesion should be used carefully when making clinical care decisions in patients with monoclonal plasma cell disorders.

Analysis of different sites within the skeleton revealed that CT was significantly superior at detecting osteolytic lesions in the axial skeleton. With some CT protocols, however, the distal portions of the humeri lay outside the field of view, so that lesions in these regions would be missed. This was particularly evident in studies in which the arms had been placed above the head with the elbows bent, as is commonly the case in examinations of the body trunk. We therefore recommend that, in WBCT protocols performed for plasma cell disorders, the arms be placed alongside the body and that the scans include both the skull up to the vertex as well as the entire femora and knees. Since beam hardening artefacts may arise if the arms are permitted to lie on the couch at the same level as the spine, thereby degrading the image quality of the vertebrae, it is likely preferable to instruct patients to fold their hands in front of the body, which places the arms in an elevated position and not at the same level as the vertebrae. Since with modern multislice CT scanners a WBCT scan lasts less than a minute, such a position is easy to maintain for vast majority of patients.

In the mentioned study from Mayo Clinic, SMM patients with PET-positive lytic bone lesions had a TTP to symptomatic disease of 21 months, as compared to 60 months for patients without lytic lesions.^14^ In the current analysis, bone destruction detected by WBCT imaging alone was likewise of prognostic significance, with a median TTP of 83 months in patients without versus 38 months in patients with lytic lesions, and a 2-year progression-free survival probability of 58% versus 33%, respectively. It cannot be entirely excluded that the presence of lytic bone lesions in WBCT could have led to an earlier initiation of treatment in some cases. We are unable to trace which events led to the diagnosis of symptomatic MM in individual cases, and have no information on whether the lytic bone lesions that had escaped detection in the CSS were actually seen in the WBCT. Since, however, the shortest TTP was longer than 2 months, we consider it unlikely that the diagnosis of symptomatic MM was immediately elicited by the results of the WBCT study.

The current findings do not answer the question of which imaging technique should be used under which conditions. While magnetic resonance imaging (MRI) has the highest sensitivity for the detection of diffuse bone marrow plasma cell infiltration, PET-CT and MRI are similarly sensitive for detecting focal lesions,^15, 16^ and WBCT (alone or done as part of PET-CT) is the best technique for detecting damage to mineralized bone.^17^ In one study, both PET-CT and MRI were shown to predict progression if they were still positive after therapy, with PET-CT being superior to MRI.^18, 19, 20^ For most clinical purposes, the CT part of a PET-CT scan can replace a separate WBCT. Nevertheless, it must be noted that this would require that the tube current to be of sufficient intensity. Often, the CT portion of PET-CT only serves for attenuation correction and is obtained with very-low-tube currents—far below that needed for true low-dose skeletal CT protocols. Although the skeleton is a high-contrast organ, they are hard to assess on such images, due to excessive image noise—and so was the case in some CT scans that were read in this study. Furthermore, some device should be used to support the arms and place them at an elevated level above the couch, since patients will not be able to maintain such position during an entire PET scan. The only significant difference between SMM patients with solely osteolyses in CT compared to others was a higher creatinine in serum reflecting presumably a more advanced stage of disease.

Since the current study was focused on comparing WBCT and CSS strictly for the detection of bone lesions, incidental findings were not a target of this study. However, Surov *et al.*^21^ were able to show 295 incidental findings in 93 MM patients, 22% of which were determined to be clinically significant. WBCT imaging is of limited value in the detection of bone marrow infiltration without frank osteolysis, especially in the cancellous bone of the spine and pelvis, because CT density at those sites is a function of both the bone marrow composition and the presence of mineralized bone trabeculae, making it difficult to determine whether the fatty bone marrow component has been replaced by a cellular myeloma infiltrate. In this setting, MRI would be the imaging technique of choice. In the long bones of adults, however, fatty conversion of the hematopoietic marrow has taken place, which makes it easy to detect abnormal cellular infiltrates, either diffuse or nodular. Nishida *et al.*^22^ have shown that infiltration of the appendicular skeleton by MM not only increased from Monoclonal Gammopathy of Undetermined Significance to SMM to MM, but that it is also of borderline prognostic significance for MM patients. Finally, although we recorded the presence of hyperdense infiltrations of the medullary cavities of the proximal long bones, analysis of these findings was not performed in the current comparative study.

In addition to being more sensitive in detecting clinical important osteolytic bone lesions compared to CSS, WBCT is also faster and more convenient for patients. However, there are some drawbacks: first the radiation dose even of modern low-dose CT protocols is ~2–4 times higher than that of at least digital CSS. Furthermore, CT is only able to display bone marrow infiltration outside the long bones because soft tissue signal is overlain by the trabecular bone in the vertebrae and the pelvis. Therefore, in this case PET and MRI have to be utilized to examine infiltration of malignant cells on regions where no bone destruction has occurred yet.^23, 24^

CT imaging has already been included in the new IMWG guidelines for the diagnosis of MM.^25^ The current study lends support to the revisions made in the revised IMWG diagnostic criteria for myeloma. Our study includes the largest number, so far, of SMM and MM patients in a comparative analysis of WBCT and CSS, confirmed not only that WBCT is significantly more sensitive than CSS for detecting myeloma-related osteolyses, but also that lytic bone lesions detected by WBCT alone are indeed prognostically significant for the progression of SMM into MM. It remains to be determined under which conditions whole-body MRI would be preferred over WBCT, a question which is beyond the scope of this study.^25^ In some circumstances MRI imaging may be needed in addition to WBCT, such as patients with SMM to detect focal lesions. In summary, we recommend the use of WBCT (alone or as part of a PET-CT protocol) instead of CSS for the evaluation of patients with suspected myeloma, and in the follow-up of patients with SMM and MM.^26^

## Figures and Tables

**Table 1:** Patient characteristics

*Parameter*	N	*%*
*Stage at time point of imaging*		
MM	146	68.8
SMM	66	31.0
		
*Serum heavy chain*		
IgA	48	23 Jan
IgD	1	0.5
IgG	131	63.0
IgG + IgA	1	0.5
IgM	1	0.5
None	26	12 May
		
*Light chain*		
Kappa	120	58.0
Lambda	82	39.4
Not determined	5	2 Apr
		
*ISS at diagnosis*		
Stage 1	89	47.6
Stage 2	69	36.9
Stage 3	29	15 May

Abbreviations: Ig, immunoglobulin; ISS, International Staging System; MM, multiple myeloma; SMM, smoldering multiple myeloma.

**Table 2:** Lytic bone lesions in CSS and WBCT, respectively, for the whole patient group

*CSS*	*WBCT*				
	*Definitely present*	*Probably present*	*Probably absent*	*Definitely absent*	*Total*
Definitely present	34	2	0	5	41
	16.0%	0.9%	0%	2.4%	19.3%
Probably present	7	0	1	6	14
	3.3%	0%	0.5%	2.8%	6.6%
Probably absent	11	4	2	10	27
	5.2%	1.9%	0.9%	4.7%	12.7%
Definitely absent	33	6	8	83	130
	15.6%	2.8%	3.8%	39.2%	61.3%
Total	85	12	11	104	212
	40.1%	5.7%	5.2%	49.1%	100.0%

Abbreviations: CSS, conventional skeletal survey; WBCT, whole-body computed tomography.

**Table 3:** Lytic bone lesions identified by CSS and WBCT, respectively, for SMM patients

*CSS*	*WBCT*				
	*Definitely present*	*Probably present*	*Probably absent*	*Definitely absent*	*Total*
Probably absent	1	1	2	5	9
	(1.9%)	(1.9%)	(3.7%)	(9.3%)	(16.7%)
Definitely absent	10	0	2	33	45
	(18.5%)	0%	(3.7%)	(61.1%)	(83.3%)
Total	11	1	4	38	54
	(20.4%)	(1.9%)	(7.4%)	(70.4%)	(100.0%)

Abbreviations: CSS, conventional skeletal survey; SMM, smoldering multiple myeloma; WBCT, whole-body computed tomography.
