# Supplementary material for: Whole-body computed tomography versus conventional skeletal survey in patients with multiple myeloma: a study of the International Myeloma Working Group
Source: Blood Cancer J. 2017 Aug 25;7(8):e599–. doi: 10.1038/bcj.2017.78 (PMC5596388; doi:10.1038/bcj.2017.78)
Supplement: Supplementary Material [file bcj201778x1.docx]

**Supplementary Information**

*Suppl. table 1: Contributing centers*

| Center | Patients fulfilling inclusion criteria | % |
| --- | --- | --- |
| Mayo Clinic Rochester | 70 | 33.0 |
| University of Athens | 43 | 20.3 |
| University of Southern Denmark Odense | 36 | 17.0 |
| University of Southern Denmark Vejle | 28 | 13.2 |
| University of Heidelberg | 17 | 8.0 |
| Kameda Medical Center | 11 | 5.2 |
| University of Liege | 7 | 3.3 |
| University of Trondheim | 0 | 0.0 |

*Suppl. table 2: Patients with/without lytic lesions by location/bone*

|  | none |  | CT only |  | CSS only |  | both |  | total |  |
| --- | --- | --- | --- | --- | --- | --- | --- | --- | --- | --- |
| Clavicle_L | 138 | (82.1%) | 20 | (11.9%) | 2 | (1.2%) | 8 | (4.8%) | 168 | (100.0%) |
| Clavicle_R | 138 | (82.1%) | 23 | (13.7%) | 0 | (0%) | 7 | (4.2%) | 168 | (100.0%) |
| CS | 167 | (79.1%) | 36 | (17.1%) | 2 | (0.9%) | 6 | (2.8%) | 211 | (100.0%) |
| Femur_L | 160 | (78.8%) | 15 | (7.4%) | 13 | (6.4%) | 15 | (7.4%) | 203 | (100.0%) |
| Femur_R | 160 | (78.0%) | 14 | (6.8%) | 13 | (6.3%) | 18 | (8.8%) | 205 | (100.0%) |
| Humerus_L | 168 | (83.2%) | 5 | (2.5%) | 12 | (5.9%) | 17 | (8.4%) | 202 | (100.0%) |
| Humerus_R | 166 | (81.8%) | 7 | (3.4%) | 14 | (6.9%) | 16 | (7.9%) | 203 | (100.0%) |
| LS | 156 | (73.6%) | 51 | (24.1%) | 3 | (1.4%) | 2 | (0.9%) | 212 | (100.0%) |
| Os_ilium | 142 | (67.0%) | 59 | (27.8%) | 0 | (0%) | 11 | (5.2%) | 212 | (100.0%) |
| Os_Ischium | 154 | (73.0%) | 38 | (18.0%) | 5 | (2.4%) | 14 | (6.6%) | 211 | (100.0%) |
| Os_pubis | 162 | (76.4%) | 30 | (14.2%) | 5 | (2.4%) | 15 | (7.1%) | 212 | (100.0%) |
| Os_sacrum | 166 | (78.3%) | 39 | (18.4%) | 1 | (0.5%) | 6 | (2.8%) | 212 | (100.0%) |
| Ribs_L | 124 | (75.6%) | 32 | (19.5%) | 2 | (1.2%) | 6 | (3.7%) | 164 | (100.0%) |
| Ribs_R | 122 | (74.4%) | 36 | (22.0%) | 2 | (1.2%) | 4 | (2.4%) | 164 | (100.0%) |
| Scapula_L | 137 | (82.0%) | 24 | (14.4%) | 1 | (0.6%) | 5 | (3.0%) | 167 | (100.0%) |
| Scapula_R | 134 | (79.8%) | 28 | (16.7%) | 0 | (0%) | 6 | (3.6%) | 168 | (100.0%) |
| Skull | 152 | (78.4%) | 19 | (9.8%) | 3 | (1.5%) | 20 | (10.3%) | 194 | (100.0%) |
| Sternum | 105 | (85.4%) | 18 | (14.6%) | 0 | (0%) | 0 | (0%) | 123 | (100.0%) |
| TS | 154 | (72.6%) | 56 | (26.4%) | 0 | (0%) | 2 | (0.9%) | 212 | (100.0%) |

*Suppl. table 3: Questionnaire for clinical parameters*

**Comparison of conventional x-ray skeletal survey and low dose computed tomography in patients with monoclonal plasma cell disorders**

Radiological Parameters

Skeletal Survey

Examination Date ___/___/___*

Skull a.p. o*

Skull sagittal o*

Upper Extremities (left humerus) o*

Upper Extremities (right humerus) o*

Cervical Spine a.p. o*

Cervical Spine sagittal o*

Thoracic Spine a.p. o*

Thoracic Spine sagittal o*

Lumbar Spine a.p. o*

Lumbar Spine sagittal o*

Pelvis a.p. o*

Lower Extremities (left femur) o*

Lower Extremities (right femur) o*

Computed Tomography Date (within 30 days)

Examination Date ___/___/___*

Transversal images o*

Coronal Reconstruction o

Sagittal Reconstruction o*

Scanner ____________________________

Collimation ____________________________

Rotation Time ____________________________

Tube Voltage ____________________________

mAs Product ____________________________

Mean Effective Dose ____________________________

Field of View (at least skull to knees) ____________________________

Slice Thickness ____________________________

Clinical Parameters

First diagnosis ___/___/___*

Prior treatment (incl. Bisphosphonates) o yes o no

If yes please specify ______________________________

______________________________

Blood/ Urin Tests (within 30 days of CT-scan)

Hemoglobin ____________________________ (g/dl)*

Calcium ____________________________ (mmol/l)*

Creatinine ____________________________ (mg/dl)*

Plasma Cells in Bone Marrow ____________________________ (%)*

o Biopsy o Smear

Albumin ____________________________ (g/l)

Beta2-Microglobulin ____________________________ (mg/l)

Cytogenetic Abnormalities (FISH) del 17p o yes o no o not tested

t(4;14) o yes o no o not tested

t(14;16) o yes o no o not tested

gain 1q21 o yes o no o not tested

Type of M-Protein:*

Heavy Chain o IgG o IgA o IgM o IgD o no

Light Chain o Kappa o Lambda o no

M-Protein in Serum ____________________________ (g/l)*

Serum Free Light Chains Kappa ____________________________ (mg/l)

Serum Free Light Chains Lambda ____________________________ (mg/l)

Kappa Light Chains in Urine ____________________________ (mg/d)*

Lambda Light Chains in Urine ____________________________ (mg/d)*

BMD (Dexa-Scan) T-Value ____________________________

Date of first progression after CT/ x-ray ___/___/___* o no progression

Date of death ___/___/___* o alive
